# Expansion of stochastic expression repertoire by tandem duplication in mouse Protocadherin-α cluster

**DOI:** 10.1038/srep06263

**Published:** 2014-09-02

**Authors:** Ryosuke Kaneko, Manabu Abe, Takahiro Hirabayashi, Arikuni Uchimura, Kenji Sakimura, Yuchio Yanagawa, Takeshi Yagi

**Affiliations:** 1 Bioresource center, Gunma University Graduate School of Medicine; 2 Department of Cellular Neurobiology, Brain Research Institute, Niigata University; 3 KOKORO-Biology Group, Laboratories for Integrated Biology, Graduate School of Frontier Biosciences, Osaka University; 4 Department of Genetic and Behavioral Neuroscience, Gunma University Graduate School of Medicine; 5 Japan Science and Technology Agency, Core Research for Evolutional Science and Technology (CREST)

## Abstract

Tandem duplications are concentrated within the Pcdh cluster throughout vertebrate evolution and as copy number variations (CNVs) in human populations, but the effects of tandem duplication in the Pcdh cluster remain elusive. To investigate the effects of tandem duplication in the Pcdh cluster, here we generated and analyzed a new line of the Pcdh cluster mutant mice. In the mutant allele, a 218-kb region containing the Pcdh-α2 to Pcdh-αc2 variable exons with their promoters was duplicated and the individual duplicated *Pcdh* isoforms can be disctinguished. The individual duplicated Pcdh-α isoforms showed diverse expression level with stochastic expression manner, even though those have an identical promoter sequence. Interestingly, the 5′-located duplicated Pcdh-αc2, which is constitutively expressed in the wild-type brain, shifted to stochastic expression accompanied by increased DNA methylation. These results demonstrate that tandem duplication in the Pcdh cluster expands the stochastic expression repertoire irrespective of sequence divergence.

Genetic variations play critical roles in animal evolution and human diseases^1^,^2^,^3^. These variations involve single nucleotide polymorphisms, small insertions/deletions, and large rearrangements, including inversions, translocations, and copy number variations (CNVs). CNVs involve either a gain (duplication) or a loss (deletion) of DNA segments. Tandem duplications, which are generated by unequal crossover, are frequent within tandemly arrayed gene clusters, which are adjacent groups of paralogous genes. Such clustered genes represent about 14% of vertebrate genes^4^ and are involved in a variety of important physiological and biochemical functions. They include, for instance, the immunoglobulin and T-cell receptor genes^5^, HOX genes^6^, zinc finger genes^7^, α– and β–globin genes^8^,^9^, and olfactory receptor genes^10^.

Although detailed analyses of these gene clusters have revealed their sophisticated gene regulation mechanisms, for example, somatic DNA rearrangements in the immunoglobulin and T-cell receptor clusters^5^, collinear expression in the HOX cluster^6^, and monoallelic and exclusive expression in the olfactory receptor cluster^10^, the evolutionary events by which these sophisticated gene regulations were acquired remain unclear.

Recently, the protocadherin (Pcdh) cluster was shown to be rich in tandem duplications^3^ and to exhibit sophisticated gene regulation mechanisms^11^,^12^. The Pcdh cluster has been identified in wide range of vertebrate species^13^,^14^,^15^. The mammalian Pcdh cluster genes are further classified into three subfamilies: Pcdh-α, Pcdh-β, and Pcdh-γ^16^. The Pcdh cluster genes probably arose by the tandem duplication and sequence divergence of existing Pcdh genes at some undetermined point in vertebrate evolution^17^,^18^. In fact, several mammalian Pcdh-α isoforms (α1-α12 in mouse and α1-α13 in human) are orthologous to four coelacanth Pcdh-α isoforms (α11–α14), and numerous mammalian Pcdh-β isoforms (β1–β22 in mouse and β1–β16 in human) are orthologous to four coelacanth Pcdh-β isoforms (β1–β4), suggesting that the common ancestors of those Pcdh-α and -β isoforms have become highly expanded in the mammalian lineage via tandem duplications^19^. The intriguing point about these expansions is that the Pcdh repertoire in mammals is more diverse than that in coelacanth. Furthermore, current human populations show a large number of CNVs in the PCDH locus^3^,^20^. However, the relevant ancestral material and human material could not be assessed, thus calling for the development of animal models of tandem duplication in the Pcdh cluster.

The diversity and sophisticated gene regulation exhibited by the Pcdh cluster genes are important for normal development of the nervous system^11^,^12^,^21^,^22^. The Pcdh cluster genes, which encode a group of diverse cadherin-related transmembrane proteins, are expressed mainly in the nervous system, and gene regulation mechanisms in the Pcdh clusters include both constitutive and stochastic expression in single neurons^23^,^24^,^25^,^26^,^27^. Gene ablation studies showed that Pcdh-α and Pcdh-γ are required for neuronal survival, synapse formation, axonal targeting, dendritic arborization, and self-avoidance of dendrites^11^,^12^,^28^,^29^,^30^. These findings led to the suggestion that the Pcdh cluster genes are likely candidates for the individualization of neurons in the vertebrate brain, which would be generated through the stochastic expression of these genes^11^,^12^,^21^,^22^. Although these findings suggest that stochastic expression in the Pcdh cluster is important in neurodevelopment, the evolutionary origin of the stochastic expression in the Pcdh cluster has remained a mystery.

To better understand the evolutionary origin of the stochastic expression and CNVs' effect in the Pcdh cluster, here we focused on the effect of tandem duplication in the mouse Pcdh-α cluster. In the present study, we engineered a targeted tandem duplication within the mouse Pcdh-α cluster, a situation somewhat comparable to that occurring during vertebrate evolution and in current human populations. The individual Pcdh-α isoforms transcribed from each duplicate exon can be distinguished in the mutant mice, enabling us to determine the manner by which their expression was regulated. The individual duplicated Pcdh-α isoforms showed diverse expression level with stochastic expression manner, even though those have an identical promoter sequence. Surprisingly, the duplicated Pcdh-αc2 isoform, which shows constitutive expression in the wild-type allele, shifted to stochastic expression accompanied by increased DNA methylation. Our results demonstrate that tandem duplication in the Pcdh cluster expands the stochastic expression repertoire irrespective of sequence divergence.

## Results

### Targeted tandem duplication in the mouse Pcdh-α cluster

**Figure 1:**
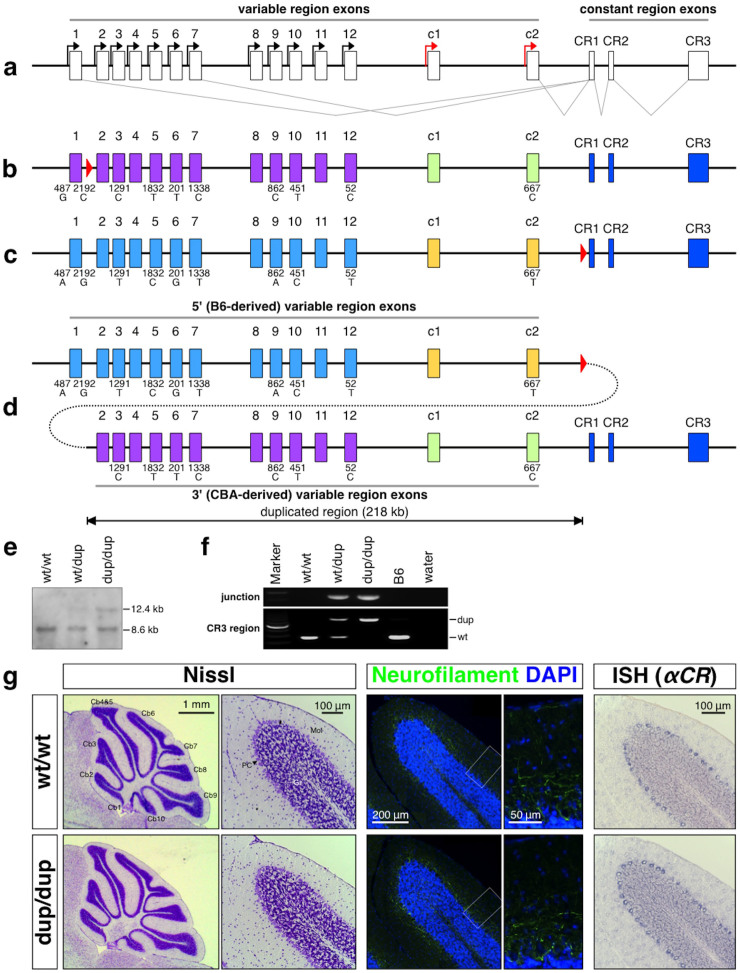
Targeted tandem duplication in the Pcdh-α cluster. (a) Genomic structure of the Pcdh-α wild-type allele. The Pcdh-α allele consists of variable region exons (1–12, c1 and c2) and constant region exons (CR1–CR3). Each variable region exon is transcribed from its own promoter in a stochastic (black arrows) or constitutive (red arrows) manner. A Pcdh-α transcript is produced from one of the variable region exons and the set of constant region exons by splicing. (b) The G16Neo allele: a loxP site was inserted between Pcdh-α1 and Pcdh-α2. The loxP site is shown as a red triangle. (c) The SR allele: a loxP site was inserted between Pcdh-αc2 and CR1. (d) The dup(2-c2) allele: duplication of Pcdh-α2-Pcdh-αc2. The dup(2-c2) allele was produced by Cre-loxP-mediated trans-allelic meiotic recombination between the G16neo and SR alleles. The duplicated segments are shown under the position of the original segments. Importantly, each duplicate Pcdh-α3, Pcdh-α5, Pcdh-α6, Pcdh-α7, Pcdh-α9, Pcdh-α10, Pcdh-α12, and Pcdh-αc2 could be distinguished by SNP analysis; the 5′ genes were from B6 and the 3′ ones were from CBA. (e & f) Confirmation of the duplication allele by Southern blotting (e) and PCR (f). G, Histological analysis of the cerebellum of 4-week-old wild-type and Pcdhα^dup(2-c2)/dup(2-c2)^ mice. Nissl staining (left), immunostaining for neurofilament (middle), and in situ hybridization using a Pcdh-α CR probe (right). Cb1-10, 1st – 10th lobule of the cerebellum; Gra, granule cell layer; Mol, molecular layer; Pur, Purkinje cell layer.

To study the consequences of tandem duplication in the Pcdh gene cluster, we generated a targeted tandem duplication in the mouse Pcdh-α cluster using the inter-strain targeted meiotic recombination (iTAMERE) system^31^. The wild-type mouse Pcdh-α cluster contains 14 large ‘variable’ exons, each of which encodes a cadherin-like type I membrane protein consisting of extracellular domains, a transmembrane domain, and a proximal cytoplasmic domain. Each variable exon is expressed from its own promoter and spliced to three short ‘constant’ exons, which encode a shared, 152-amino acid C-terminal domain (Fig. 1a)^16^. In the mutant allele [hereafter called dup(2-c2) or simply dup], a 218-kb region containing the Pcdh-α2 to Pcdh-αc2 variable exons with their promoters was duplicated; the 5′-located duplicate was derived from the C57BL/6J (B6) strain and the 3′-located one was derived from the CBA strain. We selected this particular region for duplication, because it contains both stochastically and constitutively expressed Pcdh-α isoforms, and because several of the isoforms involve a single nucleotide polymorphism (SNP) between the B6 and CBA strains in the coding region (Fig. 1b, c, d). The isoforms with a SNP between the B6 and CBA strains are Pcdh-α3, Pcdh-α5, Pcdh-α6, Pcdh-α7, Pcdh-α9, Pcdh-α10, Pcdh-α12, and Pcdh-αc2, and among these isoforms, only Pcdh-α12 has a polymorphism in its promoter region. Although the individual duplicated genes in the previous Pcdh-α duplication lines cannot be distinguished^25^, the individual duplicated genes derived from these eight Pcdh-αs in the dup(2-c2) allele can be distinguished from each other. Therefore, the dup(2-c2) mice for the first time enables the expressional analysis of the individual duplicated isoforms in the mouse Pcdh-α cluster.

Before using the iTAMERE system, we individually inserted two loxP sites into the variable region of the Pcdh-α cluster with the same orientation. First, the “G16Neo” allele^25^ was generated by inserting a loxP site between the Pcdh-α1 and Pcdh-α2 exons of the CBA allele in the TT2 ES embryonic stem (ES) cell line, which is on a CBAxB6 F1 genetic background (Fig. 1b). Second, a loxP site was inserted between the Pcdh-αc2 exon and the first exon of the constant region (CR1) in the Pcdh-α cluster to generate the “SR” allele (Fig. 1c and Supplementary Fig. S1). To insert this loxP site into the B6 allele, we used the RENKA ES cell line, which is on a pure B6 genetic background^32^.

To duplicate the sequence between the loxP site of the G16Neo allele and that of the SR allele using the Cre-loxP system, we obtained male mice that possessed the G16Neo and SR alleles (G16Neo/SR) and the Sycp1-Cre transgene, which elicits Cre recombinase expression specifically in the testis^25^. The male mice were crossed with B6 female mice, and the genotypes of the F1 pups were analyzed by PCR. The minority of F1 pups carried the dup(2-c2) allele, in which exons Pcdh-α2 to Pcdh-αc2 were duplicated (Fig. 1d), or the del(2-c2) allele, in which exons Pcdh-α2 to Pcdh-αc2 were deleted (data not shown). F1 pups carrying these duplication or deletion alleles were obtained at 5.8% (4 of 69 pups) and 1.4% (1 of 69 pups), respectively. We then analyzed the tail DNA of the duplication-containing mice by PCR to detect the Cre-mediated duplication alleles, and sequenced the PCR products to confirm the presence of the predicted junction sequences generated by the Cre-mediated site-specific recombination events. Animals homozygous for the duplicated allele (Pcdhα^dup(2-c2)/dup(2-c2)^) were obtained by crossing heterozygous (Pcdhα^wt/dup(2-c2)^) parents.

The Pcdhα^dup(2-c2)/dup(2-c2)^ pups were born with the expected Mendelian distribution (Supplementary Table S1), developed normally to adulthood, and were fertile. Histochemical analysis with Nissl staining revealed an apparently normal gross anatomy of the Pcdhα^dup(2-c2)/dup(2-c2)^ mouse brain (Fig. 1g left and Supplementary Fig. S2a). This finding was further supported by cytochrome oxidase staining showing a normal barrel structure in the Pcdhα^dup(2-c2)/dup(2-c2)^ mice (Supplementary Fig. S2a). Furthermore, neural pathway and serotonergic axon analyses by anti-neurofilament and anti-SERT staining, respectively, showed no obvious differences between the genotypes (Fig. 1g middle and Supplementary Fig. S2b). Finally, the distribution of c-fos mRNA, a well-known marker for neuronal activity, was also similar between the genotypes (Supplementary Fig. S2c). These findings suggested that the tandem duplication of exons Pcdh-α2 to Pcdh-αc2 does not result in any deleterious effects on mouse development or brain morphogenesis.

### Tandem duplication maintains the expression level of neighboring genes

**Figure 2:**
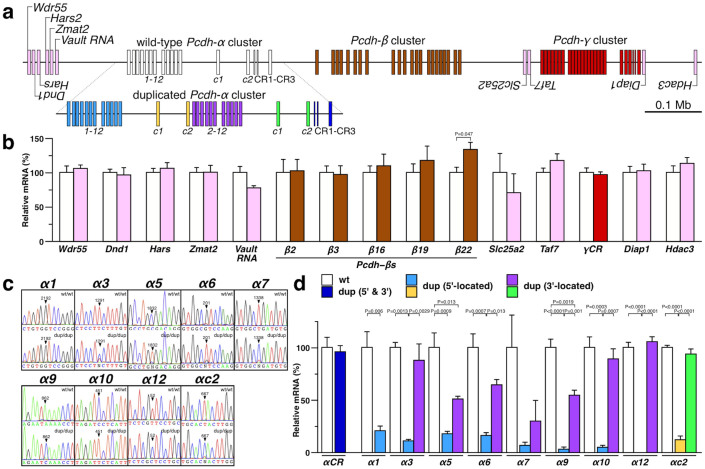
Expressional re-allocation in the duplicated Pcdh-α cluster, but no alteration in neighboring gene expression. (a) Genomic structure of the Pcdh-α cluster (upper; wild-type, lower; duplicated) and neighboring genes. (b) qRT-PCR analysis of neighboring genes in the cerebellum of 4-week-old wild-type and Pcdhα^dup(2-c2)/dup(2-c2)^ mice. wt (n = 3) and dup (n = 3). (c) SNP analysis of transcripts for Pcdh-α1, Pcdh-α3, Pcdh-α5, Pcdh-α6, Pcdh-α7, Pcdh-α9, Pcdh-α10, Pcdh-α12, and Pcdh-αc2 in the cerebellum of 4-week-old wild-type and Pcdhα^dup(2-c2)/dup(2-c2)^ mice. (d) Expression levels of wild-type and 5′- and 3′-located duplicated isoforms of Pcdh-α1, Pcdh-α3, Pcdh-α5, Pcdh-α6, Pcdh-α7, Pcdh-α9, Pcdh-α10, Pcdh-α12, and Pcdh-αc2 in the cerebellum of 4-week-old wild-type and Pcdhα^dup(2-c2)/dup(2-c2)^ mice. wt/wt (n = 3), dup/dup (n = 3).

Previous studies have indicated that tandem duplication may alter not only the expression of genes within the duplication boundaries but also of genes located in their genomic neighborhoods^33^. Prompted by these observations, we first quantified the expression levels of transcripts in the vicinity of the Pcdh-α cluster in the cerebellum of 4-week-old Pcdhα^dup(2-c2)/dup(2-c2)^ mice. The analyzed non-Pcdh genes included Wdr55, Dnd1, Hars, Zmat2, and Vault, located about 130-kb ~ 170-kb upstream from the duplication's 5′ boundary and Slc25a2, Taf7, Diap1, and Hdac3, located about 480-kb ~ 780-kb downstream from the duplication's 3′ boundary (Fig. 2a). We found that the duplication did not alter the expression of the Pcdh-β, Pcdh-γ, or non-Pcdh genes, except for a small effect on Pcdh-β22 (Fig. 2b). These results indicated that the tandem duplication of exons Pcdh-α2 to Pcdh-αc2 did not exert long-range effects on the regulation of neighboring genes.

### Tandem duplication re-allocates the manner of Pcdh-α expression

We next examined the distribution of Pcdh transcripts in the cerebellum of 4-week-old Pcdhα^dup(2-c2)/dup(2-c2)^ mice by *in situ* hybridization (ISH). We analyzed the expression of all the Pcdh-α genes (using Pcdh-αCR probe), Pcdh-α1, Pcdh-α3, Pcdh-αc2, Pcdh-β22, and all the Pcdh-γ genes (using Pcdh-γCR probe). Similar positive signals were observed for all the genes examined between the wild-type and the Pcdhα^dup(2-c2)/dup(2-c2)^ cerebellum (Fig. 1g right and Supplementary Fig. S3). These results suggested that the gene regulatory mechanisms governing the spatial distribution patterns of the Pcdh transcript were maintained in the dup(2-c2) allele.

We next examined whether both the 5′- and 3′-located duplicated Pcdh-αs were expressed. Since each duplicate of Pcdh-α3, Pcdh-α5, Pcdh-α6, Pcdh-α7, Pcdh-α9, Pcdh-α10, Pcdh-α12, and Pcdh-αc2 could be distinguished by single nucleotide polymorphism (SNP) analysis, we focused on these exons and on Pcdh-α1, which was not duplicated. We amplified the cDNA fragments of these Pcdh-α genes using specific primer combinations, and the resultant amplicons were sequenced directly (Fig. 2c). The analysis detected most of the 5′- and 3′-located duplicated Pcdh-α transcripts.

Next, the expression levels of these duplicated Pcdh-αs were quantified by qRT-PCR and cloning-mediated SNP analysis, which is highly sensitive and yields quantitative data (Fig. 2d). The expression level of the spliced CR transcripts, which are common to all the 5′-located and 3′-located duplicated Pcdh-αs, was unchanged in the Pcdhα^dup(2-c2)/dup(2-c2)^ mice. There were no significant differences in the expression levels of most of the 3′-located duplicated Pcdh-αs (Pcdh-α3, Pcdh-α6, Pcdh-α7, Pcdh-α10, Pcdh-α12, and Pcdh-αc2) compared to wild-type. In contrast, the expression levels of all the 5′-located duplicated Pcdh-αs and the 3′-located duplicated Pcdh-α5 and Pcdh-α9 genes were significantly reduced compared to wild-type. These observations revealed that the expression level of the total Pcdh-α genes was maintained, while that of individual isoforms was altered, indicating that expressional re-allocation occurred in the dup(2-c2) allele.

Interestingly, the expression levels of the 5′-located duplicated Pcdh-αs were significantly lower than those of their duplicated 3′-located counterparts. This observation suggested that, despite their identical promoter sequences, each 5′-located and 3′-located duplicated Pcdh-α receives distinct gene-regulation influences. Taken together, these findings indicate that tandem duplication alters the manner of gene regulation in the Pcdh-α cluster.

### Stochastic expression of duplicate Pcdh-α genes with identical promoter sequences

**Figure 3:**
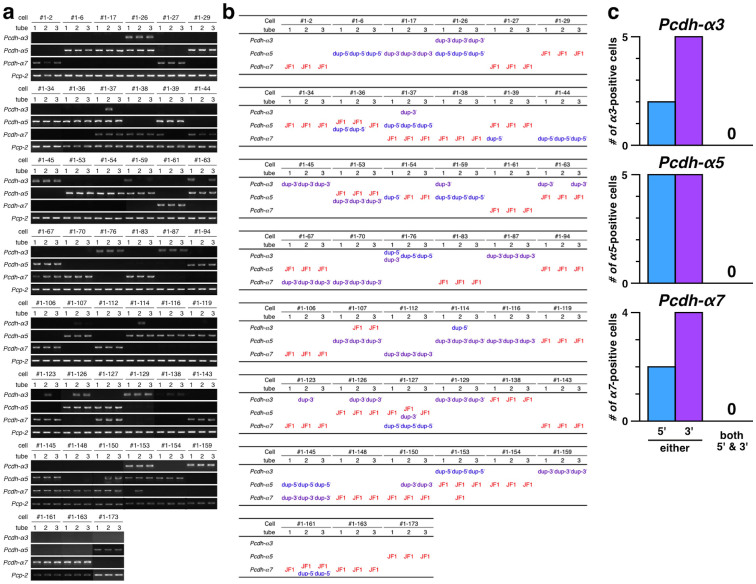
Stochastic expression of duplicated Pcdh-α3, Pcdh-α5, and Pcdh-α7 in single Purkinje cells. (a) Electrophoresis results of single-cell RT-PCR for the Pcdh-α3, Pcdh-α5, or Pcdh-α7 and Pcp-2 genes in individual Purkinje cells. The numbers #1-2 ~ #1-173 designate individual cells. 1–3, tubes into which the cDNA from an individual Purkinje cell was divided; independent PCRs were performed for each tube. This figure shows only the cells that gave PCR products for Pcdh-α3, Pcdh-α5, or Pcdh-α7. (b) SNP analysis to distinguish between Pcdh-α transcripts from the 5′-located (shown as dup-5′) and 3′-located (shown as dup-3′) isoforms on the dup(2-c2) allele and those from the wild-type allele (designated JF1). (c) Classification of Pcdh-α3, Pcdh-α5, or Pcdh-α7 expressed from the dup(2-c2) allele in individual Purkinje cells. Blue and purple bars indicate the number of Purkinje cells expressing the 5′-located and 3′-located isoforms on the dup(2-c2) allele, respectively. The cells expressing one or two of three transcripts were excluded from the analysis.

To investigate whether the duplicated Pcdh-αs retained their stochastic expression at the single-neuron level, we performed single-cell RT-PCR and SNP analysis on Purkinje cells of the Pcdhα^JF1/dup(2-c2)^ mice. The Pcdhα^JF1/dup(2-c2)^ mice were F1 mice from a JF1 × Pcdhα^dup(2-c2)/dup(2-c2)^ cross. To distinguish the 5′-located exons (B6) and 3′-located exons (CBA) in the dup(2-c2) allele, and exons in the wild-type allele (JF1) by SNP analysis, we focused on Pcdh-α3, Pcdh-α5, Pcdh-α7 (Fig. 3).

To analyze the expression of Pcdh-α3, Pcdh-α5, and Pcdh-α7 in the dup(2-c2) allele, single Purkinje cells from 4-week-old Pcdhα^JF1/dup(2-c2)^ mice were picked up by glass capillary. Complementary DNA of Pcdh-α3, Pcdh-α5, Pcdh-α7, and Pcp-2 (a marker for Purkinje cells) was synthesized from the single-cell samples in the same tube, and the resulting cDNA was then divided into three tubes and subjected to separate, first-round multiplex PCR analysis. The second round of PCR amplification was carried out individually for each tube and used nested primers for the Pcdh-α3, Pcdh-α5, Pcdh-α7 genes, and for Pcp-2. Finally, each PCR product was subjected to direct sequencing to determine from which exon the transcript was derived: i.e., the wild-type (JF1) allele or the 5′-located (dup-5′) or 3′-located (dup-3′) exons in the dup(2-c2) allele.

Of the 163 single Purkinje cells analyzed, 45 yielded PCR amplicons of Pcdh-α3, Pcdh-α5, or Pcdh-α7 from the same exons in all three tubes, and all 45 cells were positive for Pcp-2, confirming that they were differentiated Purkinje cells. In addition to three of three specific transcripts from the same exon, some cells showed one or two of three transcripts (for example, Pcdh-α3 in cell #1-37); these findings suggested that the amounts of corresponding transcripts in these cells were low, and therefore we excluded these cells from the following analysis.

For Pcdh-α3, the transcripts were derived from the wild-type exon, 5′-located exon, and 3′-located exon in 1 cell, 2 cells, and 5 cells, respectively: the wild-type (#1-138), 5′-located (#1-76, and #1-153), and 3′-located exon (#1-26, #1-45, #1-87, #1-129, and #1-159). For Pcdh-α5, the transcripts were derived from the wild-type exon, 5′-located exon, and 3′-located exon in 14 cells, 5 cells, and 5 cells, respectively: the wild-type (#1-29, #1-34, #1-36, #1-39, #1-53, #1-63, #1-67, #1-94, #1-119, #1-126, #1-127, #1-153, #1-154, and #1-173), 5′-located (#1-6, #1-26, #1-37, #1-59, and #1-145), and 3′-located exon (#1-17, #1-53, #1-107, #1-114, and #1-116). For Pcdh-α7, the transcripts were derived from the wild-type exon, 5′-located exon, and 3′-located exon in 13 cells, 2 cells, and 4 cells, respectively: the wild-type (#1-2, #1-27, #1-37, #1-38, #1-61, #1-83, #1-106, #1-123, #1-143, #1-148, #1-150, #1-161, and #1-163), 5′-located (#1-44, and #1-127), and 3′-located exon (#1-67, #1-70, #1-112, and #1-145). Only one cell expressed both the wild-type and the 3′-located exon for the same Pcdh-α isoform (Pcdh-α5 in cell #1-53) simultaneously, indicating that Pcdh-α genes are not always expressed monoallelically. Importantly, although it cannot rule out the possibility of small number of analyzed cells and possible lower expression level of the 5′-located exons may reduce the chance of detection, no cell expressed both the 5′-located and the 3′-located exon for the same Pcdh-α isoform simultaneously. These results strongly suggested that tandem duplication maintained the stochastic expression of the Pcdh-α isoforms, as seen in the wild-type brain.

### The 5′-located Pcdh-αc2 acquired stochastic expression upon tandem duplication

**Figure 4:**
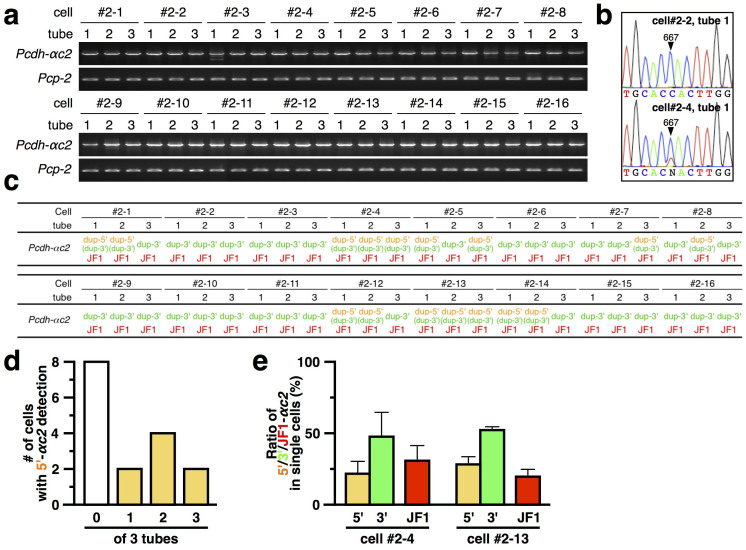
Stochastic expression of 5′-located Pcdh-αc2, but constitutive expression of 3′-located Pcdh-αc2 in single Purkinje cells. (a) Electrophoresis results of the split single-cell RT-PCR for the Pcdh-αc2 and Pcp-2 genes in individual Purkinje cells. The numbers #2-1 ~ #2-16 designate individual cells. 1–3, tubes into which the cDNA from an individual Purkinje cell was divided; independent PCRs were performed for each tube. (b) Example chromatograms showing the absence (upper) and presence (lower) of the 5′-located duplicated Pcdh-αc2 transcript. (c) SNP analysis to distinguish between Pcdh-αc2 transcripts from the 5′-located (dup-5′) and 3′-located (dup-3′) isoforms on the dup(2-c2) allele and from the wild-type allele (JF1). (d) Number of Purkinje cells showing the 5′-located duplicated Pcdh-αc2 transcript in 0, 1, 2, or 3 tubes. (e) Comparison of the Pcdh-αc2 expression levels in individual Purkinje cells (cells #2-4 and #2-13). Yellow, green, and red bars represent the expression ratio from the 5′- and 3′-located Pcdh-αc2 exons on the dup(2-c2) allele, and the wild-type (JF1) exon, respectively. Error bars indicate ±S.E.M of the averages from 3 tubes.

To investigate whether the 5′-located and 3′-located duplicated Pcdh-αc2 retained its constitutive expression at the single-neuron level, single-cell RT-PCR and SNP analysis was carried out (Fig. 4). Of the 16 single Purkinje cells analyzed, all showed the Pcdh-αc2 transcript from the wild-type (JF1) and 3′-located duplicated exon in all three tubes. They were also all positive for Pcp-2, confirming that they were differentiated Purkinje cells. These results suggested that the Pcdh-αc2 wild-type exon (JF1) and 3′-located duplicated exon were expressed constitutively in differentiated Purkinje cells.

To our surprise, the Pcdh-αc2 transcript from the 5′-located duplicated exon was detected in three tubes for only two of the 16 cells (#2-4 and #2-13). Notably, 8 of the 16 cells (#2-2, #2-3, #2-6, #2-9, #2-10, #2-11, #2-15 and #2-16) showed no Pcdh-αc2 transcript from the 5′-located duplicated exon (Fig. 4d). For those that did express it, the quantity expressed from the 5′-located duplicated exon was comparable to that from the wild-type (JF1) and 3′-located duplicated exons (Fig. 4e). These results strongly suggested that the expression of the 5′-located duplicated Pcdh-αc2 changed from constitutive to stochastic.

### Tandem duplication masks cluster-structure dependent DNA hypomethylation

**Figure 5:**
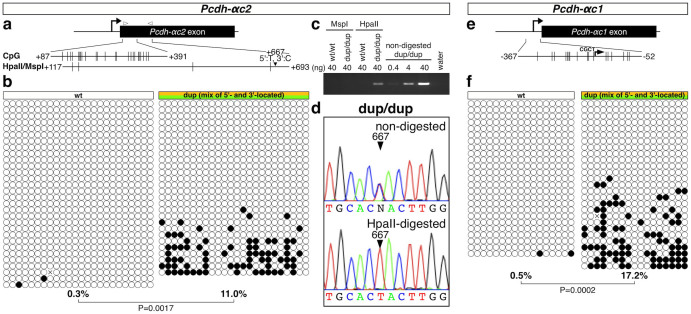
Tandem duplication increases the DNA methylation of Pcdh-αc2 and Pcdh-αc1. (a) Schematic representation of Pcdh-αc2; the positions of CpGs and HpaII/MspI sites are shown to scale by vertical lines. (b) Results of bisulfite sequencing. Each circle represents a methylated (black) or unmethylated (white) CpG dinucleotide. Each row represents a single clone. A primer set was designed to amplify the region corresponding to the 5′ region of the exon, which has the same sequence in the 5′- and 3′-located duplicated Pcdh-αc2 isoforms. The percentage below each methylation pattern indicates the CpG methylation rate for the region. C, D, Discrimination of the DNA methylation between the 5′- and 3′-located duplicated Pcdh-αc2 isoforms by HpaII digestion-mediated analysis. (c) Electrophoresis results. D, Example chromatograms showing that the HpaII-resistant fraction contained the region of the 5′-located duplicated Pcdh-αc2 exon. E, Schematic representation of Pcdh-αc1; positions of CpGs are shown to scale by vertical lines. F, Results of bisulfite sequencing. A primer set was designed to amplify the region corresponding to the Pcdh-αc1 promoter, which has the same sequence in the 5′- and 3′-located Pcdh-αc1 duplicates. The percentage below each methylation pattern indicates the CpG methylation rate for the region.

The 5′-located Pcdh-αc2 was down-regulated in the Pcdhα^dup(2-c2)/dup(2-c2)^ mouse cerebellum (Fig. 2) and acquired a stochastic expression pattern (Fig. 4), suggesting that the regulation was different between the 5′-located and the 3′-located Pcdh-αc2 in the dup(2-c2) allele. Since Pcdh-αc2 is extensively hypomethylated in the wild-type mouse brain, and higher mosaic DNA methylation levels are correlated with a lower transcription of stochastically expressed Pcdh genes^34^,^35^,^36^,^37^, we first examined the DNA methylation of Pcdh-αc2 in the Pcdhα^dup(2-c2)/dup(2-c2) ^mouse cerebellum using bisulfite sequencing (Fig. 5a and 5b). The results clearly showed higher mosaic DNA methylation of Pcdh-αc2 in the Pcdhα^dup(2-c2)/dup(2-c2)^ mouse (11.0%) compared with the wild-type mouse (0.3%).

Because bisulfite sequencing was unable to discriminate between the two Pcdh-αc2s in the dup(2-c2) allele, we further analyzed the DNA methylation using HpaII digestion-mediated DNA methylation analysis (Fig. 5c and 5d). The HpaII-resistant fraction, containing methylated CCGG, predominantly included the 5′-located duplicated Pcdh-αc2 genomic DNA. The DNA methylation level on the 5′-located duplicated Pcdh-αc2 was higher than that on the 3′-located and wild-type Pcdh-αc2.

We further examined the DNA methylation of Pcdh-αc1 in the Pcdhα^dup(2-c2)/dup(2-c2) ^mouse cerebellum. Similar to Pcdh-αc2, bisulfite sequencing showed higher DNA methylation of Pcdh-αc1 in the Pcdhα^dup(2-c2)/dup(2-c2)^ mouse (17.2%) than in the wild-type mouse (0.5%) (Fig. 5f). Taken together, these results suggested that the DNA hypomethylation in Pcdh-αc1 and Pcdh-αc2, which are constitutively expressed in the wild-type brain, is, at least in part, dependent on the cluster structure, and that tandem duplication directly increases the DNA methylation in the 5′-located Pcdh-αc1 and Pcdh-αc2.

**Figure 6:**
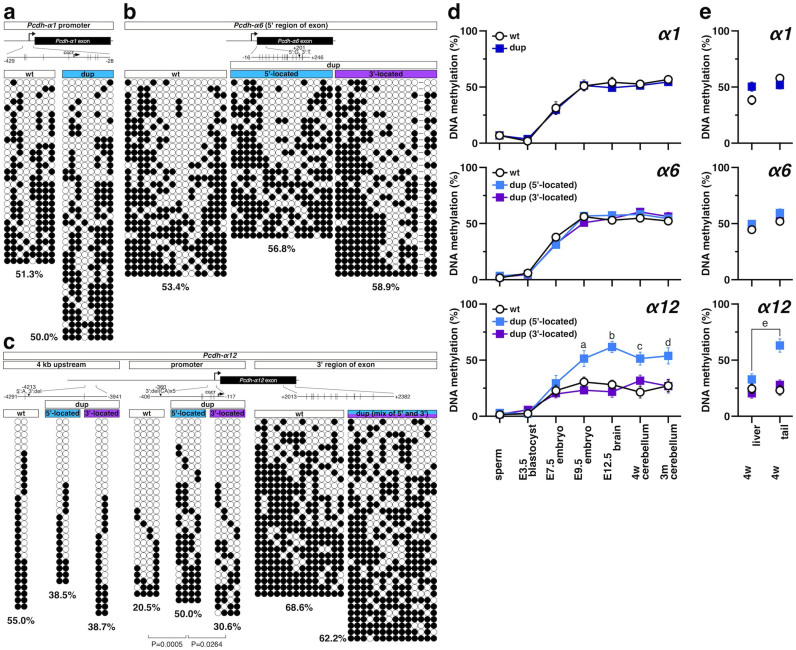
Tandem duplication increases DNA methylation of the Pcdh-α12 promoter, but not of Pcdh-α1 and Pcdh-α6. DNA methylation patterns on Pcdh-α1 (a), Pcdh-α6 (b), and Pcdh-α12 (c) in the cerebellum from 4-week-old wild-type and Pcdhα^dup(2-c2)/dup(2-c2)^ mice. (Top) Schematic representations of Pcdh-α1, 6, and 12; positions of CpGs are shown to scale by vertical lines. (bottom) Results of bisulfite sequencing. Each circle represents a methylated (black) or unmethylated (white) CpG dinucleotide. Each row represents a single clone. A primer set was designed to amplify the region corresponding to the promoter (Pcdh-α1 and Pcdh-α12) or 5′ region of the exon (Pcdh-α6) containing the SNP. In addition, the 4-kb upstream region and 3′ region of Pcdh-α12 were analyzed. The percentage below each methylation pattern indicates the CpG methylation rate for each region. (d) Changes in DNA methylation levels during development. DNA methylation levels of the Pcdh-α1 promoter (top), 5′ region of the Pcdh-α6 exon (middle), and Pcdh-α12 promoter (bottom) were analyzed by bisulfite sequencing of sperm and of mice at E3.5, E7.5, E9.5, E12.5, 4-weeks old, and 3-months old. ^a^P = 0.0071 compared with wt, P = 0.0002 compared with the 3′-located Pcdh-α12, ^b^P < 0.0001 compared with wt, P < 0.0001 compared with the 3′-located Pcdh-α12, ^c^P = 0.0005 compared with wt, P = 0.0264 compared with the 3′-located Pcdh-α12, ^d^P = 0.0167 compared with wt, P = 0.0086 compared with the 3′-located Pcdh-α12. (e) DNA methylation levels in the liver and tail of 4-week-old mice. DNA methylation levels at the Pcdh-α1 promoter (top), 5′ region of the Pcdh-α6 exon (middle), and Pcdh-α12 promoter (bottom) were analyzed by bisulfite sequencing. ^e^P = 0.0003 compared liver with tail of the 5′-located Pcdh-α12.

We next extended the DNA methylation analysis to the stochastically expressed Pcdh-α isoforms. To distinguish the 5′-located from the 3′-located exons in the dup(2-c2) allele by SNP analysis after the bisulfite reaction, we focused on the Pcdh-α1 (promoter), Pcdh-α6 (exon 5′-region), and Pcdh-α12 (promoter) (Fig. 6a–6c). A mosaic methylation pattern was observed for all three, Pcdh-α1, Pcdh-α6, and Pcdh-α12, and similar DNA methylation levels, around 50%, were observed for Pcdh-α1 and Pcdh-α6 in the wild-type and the dup(2-c2) allele. However, in the promoter region of the 5′-located Pcdh-α12, we found a higher methylation level (50.0%) than in the wild-type (20.5%) or 3′-located Pcdh-α12 (30.6%).

To identify the region with increased DNA methylation more precisely, DNA methylation around the 5′-located Pcdh-α12 promoter was examined (Fig. 6c). No significant differences in the DNA methylation level were observed for the 4-kb upstream region (38.5% ~ 55.0%, for the wild-type, the 5′-located, and 3′-located exon) or for the 3′-region exon (around 65%, for wild-type and the mixture of the 5′-located and 3′-located exon), suggesting that the increase in DNA methylation was specific for the promoter. Therefore, as in Pcdh-αc1 and Pcdh-αc2, DNA hypomethylation in the Pcdh-α12 promoter is dependent on the cluster structure, and tandem duplication directly increased the DNA methylation of the 5′-located Pcdh-α12.

To gain further insight into the cluster-dependent regulation of the DNA methylation, we next examined the methylation during development (Fig. 6d). Interestingly, while the DNA methylation level on Pcdh-α1 and Pcdh-α6 showed similar for all the loci, the DNA methylation level on the 5′-located Pcdh-α12 promoter was higher than that on the wild-type and 3′-located Pcdh-α12 promoter. The DNA methylation level on the 5′-located Pcdh-α12 resembled that of Pcdh-α1 and Pcdh-α6 throughout development. The increased DNA methylation on the 5′-located Pcdh-α12 promoter was also observed in the tail but not in the liver (Fig. 6e), suggesting that an organ-dependent DNA methylation regulator influenced the DNA methylation on the 5′-located Pcdh-α12 promoter.

Taken together, these results suggest that 3′-located genes in the wild-type Pcdh-α cluster, Pcdh-α12, Pcdh-αc1, and Pcdh-αc2, are hypomethylated in a cluster-structure dependent manner, and that these DNA hypomethylations were masked by position shift to 5′-location, which is caused by tandem duplication in the dup(2-c2) allele. This mechanism probably underlies the lower expression level of the the 5′-located Pcdh-α12 duplicate and the stochastic expression of the 5′-located Pcdh-αc2 duplicate in the dup(2-c2) allele.

## Discussion

**Figure 7:**
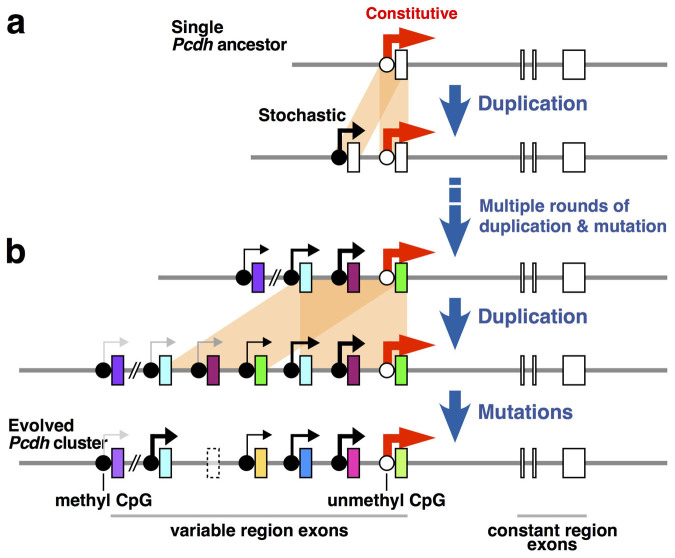
Model for the expansion of stochastic Pcdh-α expression via tandem duplication. (a) Hypothetical evolutionary history from an ancestral single Pcdh gene. (b) Hypothetical evolutionary history from clustered Pcdh-α genes. Tandem duplication (orange shading) creates duplicated exons in the Pcdh-α cluster. Both of the duplicate stochastically expressed Pcdh-α isoforms (those with black-grey arrows) retain their stochastic expression. In contrast, from constitutively expressed isoforms (thick red arrow), the 3′-located duplicated Pcdh-α maintains its constitutive expression, but the 5′-located one shifts to stochastic expression accompanied by higher DNA methylation. Mutations in exons and/or regulatory sequences generate diverse coding exons, pseudogenes, and altered gene regulatory systems. Boxes, Pcdh-α exons. Dotted box, pseudogene.

Tandem duplications are concentrated within the Pcdh cluster throughout vertebrate evolution and as CNVs in human populations^3^,^19^,^20^, but the effects of tandem duplication in the Pcdh cluster remain elusive. Here we revealed a critical role for tandem duplication in the Pcdh cluster gene regulation. The individual duplicated Pcdh-α isoforms showed diverse expression level with stochastic expression manner, even though those have an identical promoter sequence. Interestingly, the 5′-located duplicated Pcdh-αc2, which is constitutively expressed in the wild-type brain, shifted to stochastic expression upon tandem duplication, accompanied by increased DNA methylation. These observations suggest that tandem duplication has been beneficial for the acquisition of the stochastic expression and the expansion of its repertoire through vertebrate evolution and in human populations (Fig. 7).

What are the mechanisms by which individual duplicated Pcdh-αs, each of which has an identical promoter sequence, are expressed stochastically? The present data are consistent with our previous findings, which is that the stochastic expression of Pcdh-α cluster genes is governed by enhancer and promoter DNA methylation^25^,^38^. The enhancers for the Pcdh-α genes, named HS5-1 and HS7, are located downstream of the Pcdh-α cluster^39^,^40^,^41^, and the present data suggest that the HS5-1 and/or HS7 enhancers in the duplicated allele are still effective at even greater distances than in the wild-type allele (distance between the HS5-1 enhancer and the most distal promoter (Pcdh-α1) in the wild-type allele was ~280 kb; in the dup(2-c2) allele, it was ~500 kb). However, the present finding of lower expression of the 5′-located duplicated Pcdh-α genes than the 3′-located duplicated Pcdh-α genes in the dup(2-c2) allele indicates that the longer distance between the HS5-1 enhancer and the promoter lowers the probability of expression. The present data strongly support the “enhancer sharing and stochastic promoter competition” model, in which a single enhancer stochastically governs the expression of the Pcdh cluster genes^25^.

Stochastic promoter competition has also been suggested for other gene clusters, such as the olfactory receptor MOR28 cluster^10^,^42^ and the primate red and green-pigment genes^43^. Furthermore, non-stochastic promoter competition has been suggested for other gene clusters, such as the Hoxd gene cluster^44^, α– and β–globin gene cluster^8^,^9^, and zebrafish red opsin genes^45^; these gene clusters show temporally and spatially organized expression. Thus, promoter competition is widely distributed through gene clusters. We argue that the characteristics of the enhancer and/or promoter add further sophistication to gene regulatory systems.

Here we found that the 5′-located duplicated Pcdh-αc2, which is constitutively expressed in the wild-type brain, acquired stochastic expression upon tandem duplication, accompanied by increased DNA methylation. These results provide supportive evidence for previous findings that mosaic DNA methylation states are correlated with the stochastic expression of Pcdh-α isoforms in wild-type mouse brain^36^. Previous studies described the suppression of promoter DNA methylation, which locates the region 3′ proximal to the Pcdh-α cluster (Pcdh-α12, Pcdh-αc1 and Pcdh-αc2 in wild-type mouse brain and PCDHA13, PCDHAC1, and PCDHAC2 in normal human kidney and human kidney tumor)^34^,^36^,^38^. Our recent results suggested that the establishment of mosaic DNA methylation patterns in the Pcdh clusters is cooperatively regulated by the specificity of Dnmt3b, the gene cluster structure, the enhancer element, and the sequence features^38^. Another possible mechanism is an altered enhancer-promoter interaction^40^,^46^. Collectively, the mechanism underlying the shift of the 5′-located duplicated Pcdh-αc2 to stochastic expression is an important question for future study.

The experiments described here reveal some role of tandem duplication on gene regulatory differentiation, that include expression level divergences of the duplicated Pcdh-α genes and changes from constitutive to stochastic manner of the 5′-located duplicated Pcdh-αc2 gene. The data suggest that the current state of the Pcdh-α cluster in mammals, in terms of how it is expressed, was shaped by tandem duplication and distance from promoter to enhancer. Furthermore, although it is not clear that Pcdh-α gene number or stochastic expression have been strongly selected during vertebrate evolution, the present results may imply the evolutionary history of Pcdh cluster gene regulation. Previous reports have suggested that Pcdh cluster evolution included successive tandem duplications and sequence divergences^16^,^17^,^19^. Here, we propose a model in which stochastic expression of the duplicated Pcdh genes is immediately acquired after tandem duplication, which precedes sequence divergence (Fig. 7).

The human PCDH cluster is particularly rich in CNVs, including duplications and deletions^3^,^20^,^47^ (see Database of Genome Variants: http://dgv.tcag.ca/gb2/gbrowse/dgv2_hg18/?name=chr5:140050001..141050000). The present study showed that the tandem duplication resulted in healthy mice with a macroscopically normal brain. This result can be explained in part by the maintenance of the expression levels and distribution patterns of the total Pcdh-α transcript, by the re-allocation of Pcdh-α isoform expression, and by the maintenance of the expression levels of neighboring genes, upon duplication. Thus, it is likely that following various CNV events in the human PCDH cluster, the total expression level, dual gene-regulatory mechanisms, and stochastic expression of the human PCDH genes are maintained. This robustness may provide the predominant reason for the frequent CNVs in the human PCDH cluster. One study reported that there is no phenotypic link between a CNV in the human PCDH cluster, a 16.7-kb deletion affecting PCDHA8-A10, and psychiatric disorders^47^. Recently, a *de novo* gene disruption in PCDHA13 was reported in autism^48^. Furthermore, Anitha A. *et al.* reported strong genetic evidence of PCDHA as a potential candidate gene for autism^49^. The PCDHA cluster is also a candidate locus for bipolar disorder^50^. Furthermore, deletion of PCDHA1-PCDHA9 is associated with higher brain function, such as music perception^51^. It will be interesting to investigate the effects of these genetic mutations on the PCDHA expression and neural circuit formation.

Recent human genome analyses revealed that tandem duplications contribute to human phenotypes, including many psychiatric disorders, color vision, Parkinson's disease, and Rheumatoid arthritis^1^,^3^,^52^. For example, duplications of 7q36.3, which contains the vasoactive intestinal peptide receptor gene VIPR2, confer significant risk for schizophrenia, and VIPR2 mRNA levels are increased differently among duplication carriers^53^. These findings suggest the importance of conducting detailed investigations addressing the effects of duplications on gene regulation. The iTAMERE approach enables careful analyses directed toward understanding the etiology of CNV-associated human disorders.

The vertebrate Pcdh cluster shows remarkable similarity to the Drosophila Dscam1 gene, within which tandem duplication is frequent throughout its evolutionary history^54^. Importantly, recent studies demonstrated that the stochastic gene regulation in Pcdh and Dscam1 play important roles in neural circuit development by providing a source for cell surface diversity^11^,^12^. These findings suggest essential roles for tandem duplications in the evolution of vertebrate and invertebrate nervous systems. Further studies aimed at dissecting fine-scale neural circuits in the Pcdhα^dup(2-c2)/dup(2-c2)^ mice will improve our understanding how tandem duplication in the Pcdhα cluster contribute to brain function.

## Methods

### Animals

B6 mice were purchased from Charles River Japan. The wild-type mouse strain JF1 was obtained from the National Institute for Genetics (Mishima, Shizuoka, Japan). All animals were maintained in a specific pathogen-free space under a 12-h light/dark regimen. Experimental procedures were performed in accordance with the Guide for the Care and Use of Laboratory Animals of the Science Council of Japan and approved by the Animal Experiment Committee of Gunma University and Osaka University.

### Generation of Pcdhα^dup(2-c2)/dup(2-c2)^ mice

By crossing SR mice (see Supplemental Experimental Procedures) with Sycp1-Cre transgenic mice^25^, we generated SR mice carrying the Sycp1-Cre transgene. We crossed these mice with mice bearing the G16Neo allele, then selected male offspring bearing the SR allele, G16Neo allele, and the Sycp1-Cre transgene (Pcdhα^SR/G16Neo^, Sycp1-Cre). We then crossed Pcdhα^SR/G16Neo^, Sycp1-Cre male and B6 female, and genotyped the pups using genomic DNA extracted from the tail. Some of these pups carried the duplicated [dup(2-c2)] or deleted [del(2-c2)] allele as a result of TAMERE in the testis^31^. To identify the Pcdhα^wt/dup(2-c2)^ mice, we performed Southern blotting and PCR analyses (Fig. 1e and 1f). The BamHI-digested genomic DNA from the tail was subjected to Southern blotting using probe A (the same probe used to identify the SR allele). A band at 8.6 kb indicated the wild-type allele, and a band at 12.4 kb indicated the dup(2-c2) allele. Pcdhα^dup(2-c2)/dup(2-c2)^ mice were obtained by crossing Pcdhα^wt/dup(2-c2)^ parents, which were backcrossed with B6 for more than three generations.

### *In situ* hybridization

*In situ* hybridization (ISH) was performed essentially as described previously^27^. The details are provided in the Supplemental Experimental Procedures.

### Expression analysis in cerebellum

The cerebellum was dissected from 4-week-old mice and immediately frozen in liquid nitrogen. The tissue was homogenized with a Polytron homogenizer. Total RNA was isolated using RNeasy (Qiagen), according to the supplier's recommendations. To obtain cDNA, 2.5 μg of the total RNA was treated with DNase I (Takara) and reverse transcribed with SuperscriptIII reverse transcriptase (Invitrogen) using random primers in a 40-μl reaction volume.

For the SNP analyses, PCR for each Pcdh-α isoform was performed using 0.4 μl of cDNA from the cerebellum of a 4-week-old mouse as a template. The primer sequences used for the SNP analyses are shown in Supplementary Table S2. For direct SNP analyses, the PCR products were sequenced using a standard method. For the cloning-mediated SNP analysis, the PCR products were cloned into pT7-Blue (Novagen), white colonies were randomly picked, and individual clones were sequenced using a standard method. The SNPs used for the direct sequencing analysis are shown in Supplementary Table S3.

The qRT-PCR was performed with SYBR Premix ExTaq II (Takara) using the 7500Fast Real-Time PCR system (Applied Biosystems) or LightCycler480 (Roche). The primer sequences used for qRT-PCR are shown in Supplementary Table S2. All data shown are normalized to beta 2 microglobulin. The efficiency of all the primer pairs was confirmed by performing reactions with serially diluted samples. The specificity of all the primer pairs was confirmed by analyzing the dissociation curve.

### Split single-cell RT-PCR

Single-cell RT-PCR was performed essentially as described previously, with small modifications^24^,^55^. The details are provided in the Supplemental Experimental Procedures.

### DNA methylation analysis by bisulfite sequencing and HpaII digestion

The genomic DNA was prepared with the QIAamp DNA Micro Kit (Qiagen) from the cerebellum of a 4-week-old or 3-month-old mouse, or from sperm, or with the EpiTect Plus LyseAll Kit (Qiagen) from the brain of an E12.5 embryo, head of an E9.5 embryo, E7.5 whole embryo, or blastocyst (E3.5), according to the supplier's recommendations. Sperm was obtained from the cauda epididymides of adult male mice. Embryos were obtained by the natural breeding of Pcdhα^wt/dup(2-c2)^ parents. The morning of the vaginal plug was designated E0.5. Bisulfite conversion was performed with the EpiTect Plus DNA Bisulfite Kit (Qiagen), according to the supplier's recommendations. We used “MethPrimer” (http://www.urogene.org/methprimer/) to design primers for use on bisulfite-treated DNA^56^. The primers used for DNA amplification are listed in Supplementary Table S2. The first PCR program consisted of 95°C for 3 min, 25 cycles of 95°C for 30 s, 60°C for 30 s, 72°C for 30 s, and a final extension of 72°C for 7 min. The second PCR was carried out using the same program as the first, except that 32 cycles were performed. To obtain the methylation profile from the acquired data, we used the web-based tool, “QUMA” (http://quma.cdb.riken.jp/)^57^.

In the HpaII digestion-mediated DNA methylation analysis, HpaII was used to distinguish between methylated (undigested) and unmethylated (digested) HpaII/MspI sites, whereas MspI digested both methylated and unmethylated HpaII/MspI sites. The HpaII/MspI-digested DNA was subjected to PCR analysis to amplify the SNP-containing regions. We mixed 500 ng of DNA with 10 U HpaII or 10 U MspI, and restriction buffer in a 10-μl reaction volume. Samples were incubated at 37°C overnight, and we used 40 ng of the digested DNA for the PCR reaction. The primers are listed in Supplementary Table S2. The PCR program consisted of 95°C for 3 min, 30 cycles of 95°C for 30 s, 60°C for 30 s, 72°C for 1 min, and a final extension of 72°C for 7 min. The PCR products were sequenced using a standard method. The results shown are representative of two independent experiments.

### Statistical analysis

Statistical analysis was performed using GraphPad Prism Version 6.0 (GraphPad Software, La Jolla, CA, USA) and was performed using one-way ANOVA followed by Bonferroni's post hoc test, or using an unpaired two-tailed Student's t test if applicable. All data are expressed as the mean ± S.E.M.

## Author Contributions

R.K. and T.Y. conceived and designed the experiments, analyzed the data and wrote the manuscript. R.K. and M.A. performed research; R.K., M.A., T.H., A.U., K.S. and Y.Y. contributed unpublished reagents/analytic tools.

## Supplementary Material

Supplementary Information Supplementary information
