# Supplementary material for: Expansion of stochastic expression repertoire by tandem duplication in mouse Protocadherin-α cluster
Source: Sci Rep. 2014 Sep 2;4:6263. doi: 10.1038/srep06263 (PMC4151104; doi:10.1038/srep06263)
Supplement: Supplementary Information [file srep06263-s1.pdf]

Expansion of stochastic expression repertoire by tandem duplication in mouse Protocadherin-α cluster

Ryosuke Kaneko[1], Manabu Abe[2], Takahiro Hirabayashi[3], Arikuni Uchimura[3], Kenji Sakimura[2], Yuchio Yanagawa[1,4,5] & Takeshi Yagi[3,5]*

[1]Bioresource center, Gunma University Graduate School of Medicine
[2]Department of Cellular Neurobiology, Brain Research Institute, Niigata University
[3]KOKORO-Biology Group, Laboratories for Integrated Biology, Graduate School of Frontier Biosciences, Osaka University
[4]Department of Genetic and Behavioral Neuroscience, Gunma University Graduate School of Medicine
[5]Japan Science and Technology Agency, Core Research for Evolutional Science and Technology (CREST)

Supplementary information

Contents:

Supplemental Experimental Procedures 1
Supplementary Figures 6
Supplementary Tables 1-3 9
Supplemental References 15

Supplemental Experimental Procedures

Generation of the SR targeting mouse

To generate the SR targeting vector, we used the Red recombination system [1]. The *E. coli* strain EL350, into which the purified mouse BAC RP23-318M13 was transferred, was used to generate the SR targeting vector. The targeting vector used in the BAC modification was a pPE7neoW-F2LF plasmid containing an *frt*-flanked *neo*^{r} cassette, which was obtained from Junji Takeda (Osaka University). First, to introduce a *loxP* site into the 3' region of HS5-1 in the BAC, the homologous fragments for the BAC recombineering were inserted into the

pPE7neoW-F2LF plasmid. These homology arms were generated by PCR amplification of the BAC. The 5' homology fragments, which were amplified by the SRC-F and SRC-R primers, were subcloned into the *Eco*RI restriction site, and the 3' homology fragments, which were amplified by the C1D-F and C1D-R primers, were subcloned into the *Bam*HI and NotI restriction site. The primer sequences used are shown in Table S1. Next, the loxP-added SR fragments, which were amplified by the SRD-F and SRD-R primers, were subcloned into the *Bam*HI restriction site. The floxed neo^r gene with homology arms and SR fragments was excised by *Xho*I digestion, and gel-purified. The purified neo^r cassette (800 μg) was electroporated into EL350 cells, which had been induced for the Red recombination function by prior growth at 42 °C for 15 min. Transformants were selected on Kanamycin-containing plates. The modified BACs were verified by PCR.

Next, by homologous recombination, BAC DNA fragments containing the floxed SR and neo^r gene were inserted into the pBRSDT vector, which contained the gene for diphtheria toxin A (DT-A) in the *Sal*I site of pBR322 [2]. The homology fragments for this recombination were amplified by the C1H-F and C1H-R primers and the C1F-F and C1F-R primers, and were subcloned into pBRSDT at the *Hin*dIII and *Nhe*I I restriction sites. We retrieved the 14.5-kb BAC DNA fragments that contained the floxed SR and *frt*-flanked neo^r genes, and inserted them into pBRSDT. The retrieved fragments were used as a targeting vector.

The SR targeting vector (5 nM), which was linearized by NotI, was electroporated into B6-RENKA ES cells (Fig. S1) [3]. Colonies were selected in G418-containing medium. The colonies were screened for homologous recombinants by Southern hybridization. The targeted ES clones were identified by Southern blotting analysis using three probes: Probe A, which was designed to hybridize with the region amplified with CP1proA-F and CP1proA-R; Probe B, which was designed to hybridize with the region amplified with C1proB-F and C1proB-R; and Neo, which encoded the PGK-neo gene. Recombinant ES cell clones were injected into ICR 8-cell-stage embryos, and the male chimeras were bred with B6 mice. To delete the *frt*-flanked neo^r gene in the SR targeting allele, these offspring were crossed with FLP66 transgenic mice [4].

Genotyping of mutant mice

The genotyping of mutant mice was performed by PCR analysis of the genomic DNA. PCRs were performed at 95°C for 3 min, 35 cycles of 95°C for 30 sec, 60°C for 30 sec, 72°C for 1 min; then 72°C for 7 min, unless otherwise stated. For Sycp1-Cre mice, the Cre-F and Cre-R primers were used. For G16Neo mice, the following primers were used: G16F and G16R1 for

the wild-type allele, and G16F and G16R2 for the G16Neo allele. For SR mice, the SR134F and SR454R primers were used. To detect the dup(2-c2) allele, the SR202F and IR/DR1.0F primers were used. To distinguish among wild-type, heterozygous, and homozygous for the dup(2-c2) allele, the following primers were used: CNR3376F and CNR4077R (PCR was performed at 95°C for 3 min, 35 cycles of 95°C for 30 sec, 55°C for 30 sec, 72°C for 1 min; then 72°C for 7 min), followed by TaqI digestion of the PCR product at 55°C.

Split single-cell RT-PCR and SNP analysis

Single-cell RT-PCR was performed essentially as described previously, with small modifications [5-7]. The primer sets and sequences are shown in Supplemental Table S1. In brief, Purkinje cells were prepared from the cerebellum of 4-week-old mice, from the F1 litter of a Pcdh$\alpha^{dup(2\text{-}c2)/dup(2\text{-}c2)}$ female x JF1 male. The tissue was dissected and digested with 90 units of papain (Worthington) at 37°C for 30 min in 10 ml of dissociation solution [0.002% DL-cysteine HCl (Sigma), 0.05% DNase I (Sigma), 0.1% bovine serum albumin (Sigma), and 0.05% glucose (Nacalai Tesque)]. The digested tissue was spun for 8 min at 300 x g, and the pellet was resuspended in Dulbecco's modified Eagle's medium (Sigma). To remove debris, the cells were filtered through a 100-μm cell strainer (Falcon). Single Purkinje cells were picked up by glass capillary and placed in thin, 200-μl PCR tubes, with 6 μl of RNase-free water. Complementary DNA was synthesized from the single-cell samples after adding 2.7 pmol/reaction of Pcdh-α and *Pcp-2* RT primer in a total volume of 10 μl, using 6 units of PrimeScript reverse transcriptase (Toyobo, Japan), according to the manufacturer's protocol, at 42°C for 60 min. The reaction was stopped by heating at 70°C for 15 min.

The cDNAs derived from a single Purkinje cell were split into three PCR tubes as 3.3-μl aliquots and used as a template for the first round of a multiplex/duplex PCR, which was performed using the 3.3 μl of cDNA, 2.5 μl of 10x Ex-taq PCR buffer, 2.5 μl of 2.5 mM each dNTP mix, 1.5 μl of the first PCR primer set, 0.1 μl of Ex-taq HS polymerase (Takara), and 15.1 μl of water. The PCR conditions were an initial 3 min at 95°C, then 5 cycles of 20 sec at 95°C, 2 min at 72°C, then 5 cycles of 20 sec at 95°C, 2 min at 70°C, then 5 cycles of 20 sec at 95°C, 2 min at 68°C, and then 20 cycles of 20 sec at 95°C, 2 min at 65°C and 7 min at 72°C. The first PCR products were split into 0.1-μl aliquots, which were used as the template in the second round of nested PCR with 2 μl of 10x Ex-taq PCR buffer, 2 μl of 2.5 mM each dNTP mix, 0.4 μl of the second PCR primer set, 0.1 μl of Ex-taq HS polymerase (Takara), and 15.4 μl of water. The PCR conditions for the *Pcdh-β* genes were an initial 3 min at 95°C, 35 cycles of

30 sec at 95°C, 30 sec at 65°C, and 1 min at 72°C, and then 7 min at 72°C.

The secondary PCR products were divided into two tubes. Half of the PCR-amplified product was analyzed by agarose gel electrophoresis. The other half was purified by ExoSAP-IT (GE Healthcare) and sequenced by direct sequencing, using a BigDye^{TM} DNA sequencing kit (version 3.1) (ABI). The data were analyzed on an ABI Prism 3130 Genetic Analyzer. Direct sequencing permitted us to identify the exon from which each purified PCR product was derived.

To analyze the expressional ratio of 5'/3'/JF1-*Pcdh-αc2* in single Purkinje cells, the 2nd PCR products were cloned into pT7-Blue (Novagen), white colonies were randomly picked, and individual clones were sequenced with the C2+542F primer using a standard method.

In situ hybridization

In situ hybridization was performed as described previously [6, 8, 9] on 10-μm-thick frozen sagittal sections prepared from wild-type and Pcdhα$^{dup(2\text{-}c2)/dup(2\text{-}c2)}$ mice using digoxigenin (DIG)-labeled cRNA probes. The mouse ages and regions examined are indicated in the figure legends. The probes for the *Pcdh* genes were the same as in our previous studies [6, 10, 11]. The probe for *c-fos* was the same as that used by Takahata *et al* [12].

To obtain tissue samples, mice were deeply anesthetized with diethyl ether, then the brain was removed, embedded in O.C.T. compound (Sakura), and quickly frozen in isopentane cooled with dry ice. Sections (10-μm thick) were cut on a cryostat (Leica CM3050), thaw-mounted on slides (Matsunami), and air-dried. The sections were fixed in 4% paraformaldehyde (PFA) in 0.1 M phosphate buffer (PB, pH 7.3) for 10 min, washed three times in PBS, pH 7.4, acetylated for 10 min in 0.25% acetic anhydride in 0.1 M triethanolamine-HCl, pH 8.0, and washed three more times with PBS. Prehybridization was performed with hybridization buffer [50% formamide, 5xSSC (20x SSC is 3 M NaCl, 0.3 M sodium citrate, pH 7.0), 5x Denhardt's, 250 μg/ml yeast tRNA, 500 μg/ml salmon sperm DNA, and 0.2% RNasin RNase inhibitor (Promega)] for 30 min. The DIG-labeled cRNA probes were denatured for 5 min at 82°C and chilled on ice. The sections were covered with hybridization buffer containing 1 μg/ml of the DIG-labeled cRNA probes, added dropwise, and a coverslip was added. The slides were incubated for 12 h at 72°C in a humidified chamber (50% formaldehyde, 5x SSC), washed three times with 0.2x SSC at 72°C, washed three times with TBS, pH 7.5 (100 mM Tris-HCl, pH 7.5, 100 mM NaCl), and rinsed with TNT (0.05% Tween 20 in TBS, pH 7.5). To detect the hybridized probes, the sections were blocked with 1x blocking solution (Roche) in TNT for 30

min, then incubated with alkaline phosphatase (AP)-conjugated anti-digoxigenin antibody (1:1000 dilution, Roche) in the blocking solution for 1 h. The sections were rinsed three times with TBS, pH 7.5, and the enzymatic activity was visualized with 0.2 mM 5-bromo-4-chloro-3-indolyl-phosphate, 0.2 mM nitro blue tetrazolium (NBT/BCIP) in 100 mM Tris-HCl, pH 9.5, 100 mM NaCl, 20 mM $MgCl_2$, in the dark, until the signal reached a satisfactory intensity.

For other histological analyses, mice were anesthetized and perfused with phosphate-buffered saline (PBS) followed by 4% paraformaldehyde in PBS. After perfusion, the brain was rapidly removed and then immersed in the same fixative for 2 hr. The tissues were immersed in PBS containing 25% sucrose overnight for cryoprotection. Sections (20-μm thick, unless otherwise stated) were cut on a cryostat and were rinsed with 0.1 M phosphate buffer. Sections were treated with anti-neurofilament (clone 2H3; Developmental Studies Hybridoma Bank), or anti-SERT (rabbit, Frontier Institute, Japan), and then with Alexa 488-conjugated anti-mouse or Alexa 594-conjugated anti-rabbit IgG (Molecular Probes) diluted in PBS containing 5% normal goat serum, 2% bovine serum albumin, and 0.2% Triton X100, for 1 h. Finally, the sections were stained with DAPI and coverslipped with CC/Mount (Diagnostic BioSystems). For Nissl staining, the sections were stained with cresyl violet. For cytochrome oxidase staining, the sections (50-μm thick) were incubated in a solution containing 0.05% cytochrome c, 0.05% diaminobenzidine, and 4% sucrose for 3 h at 37 °C. The sections were then rinsed with 0.1 M phosphate buffer and mounted onto glass slides.

Supplementary Figures

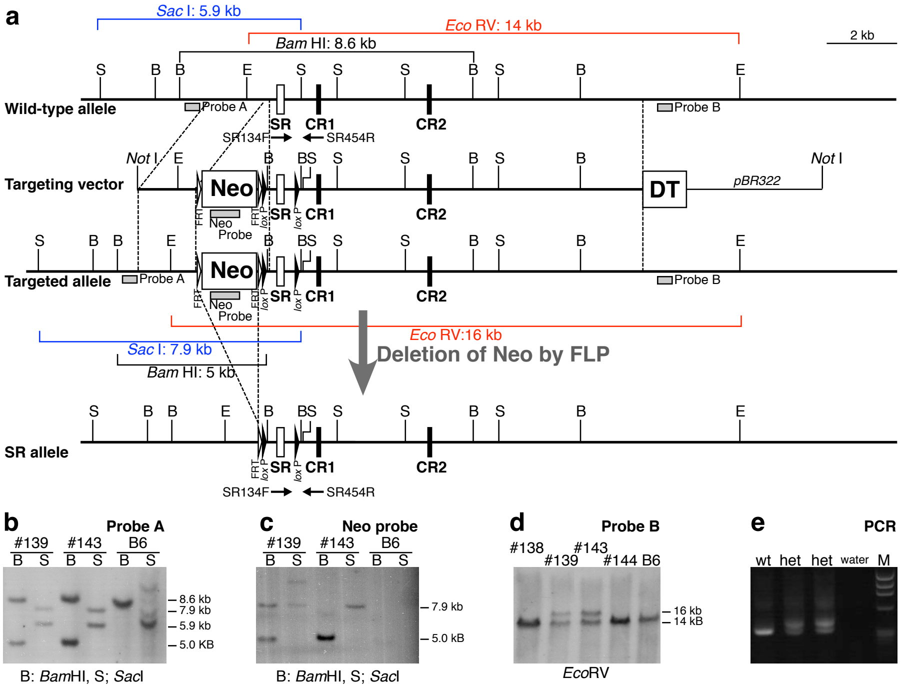

Figure S1. Generation of the SR allele. (a) Schematic diagram of the wild-type, SR targeting vector, targeted, and SR alleles. Filled triangles represent *loxP* sites. Open triangles represent *frt* sites. B, *Bam*HI; E, *Eco*RV; S, *Sac*I. (b) Southern blotting of homologous recombinant ES cell DNA digested by *Bam*HI and *Sac*I with Probe A, shown in Figure S1a. In the *Bam*HI-digested lane, the band at 8.6 kb indicates the wild-type allele, and the band at 5.0 kb indicates the SR floxed allele. In the *Sac*I-digested lane, the band at 5.9 kb indicates the wild-type allele, and the band at 7.9 kb indicates the SR floxed allele. (c) Southern blotting of homologous recombinant ES cell DNA digested by *Bam*HI and *Sac*I with Probe Neo, shown in Figure S1a. In the *Bam*HI-digested lane, the band at 5.0 kb indicates the SR floxed allele. In the *Sac*I-digested lane, the band at 7.9 kb indicates the SR floxed allele. (d) Southern blotting of homologous recombinant ES cell DNA digested by *Eco*RV with Probe B. The band at 14 kb indicates the wild-type allele, and the band at 16 kb indicates the γHS4-5Neo allele. The ES clone #143 was used to generate chimera mice. (e) Genotyping of the SR allele by PCR using genome DNA from tail as a template with the SR134F and SR454R primers. M, markers (ϕX174 HaeIII digest).

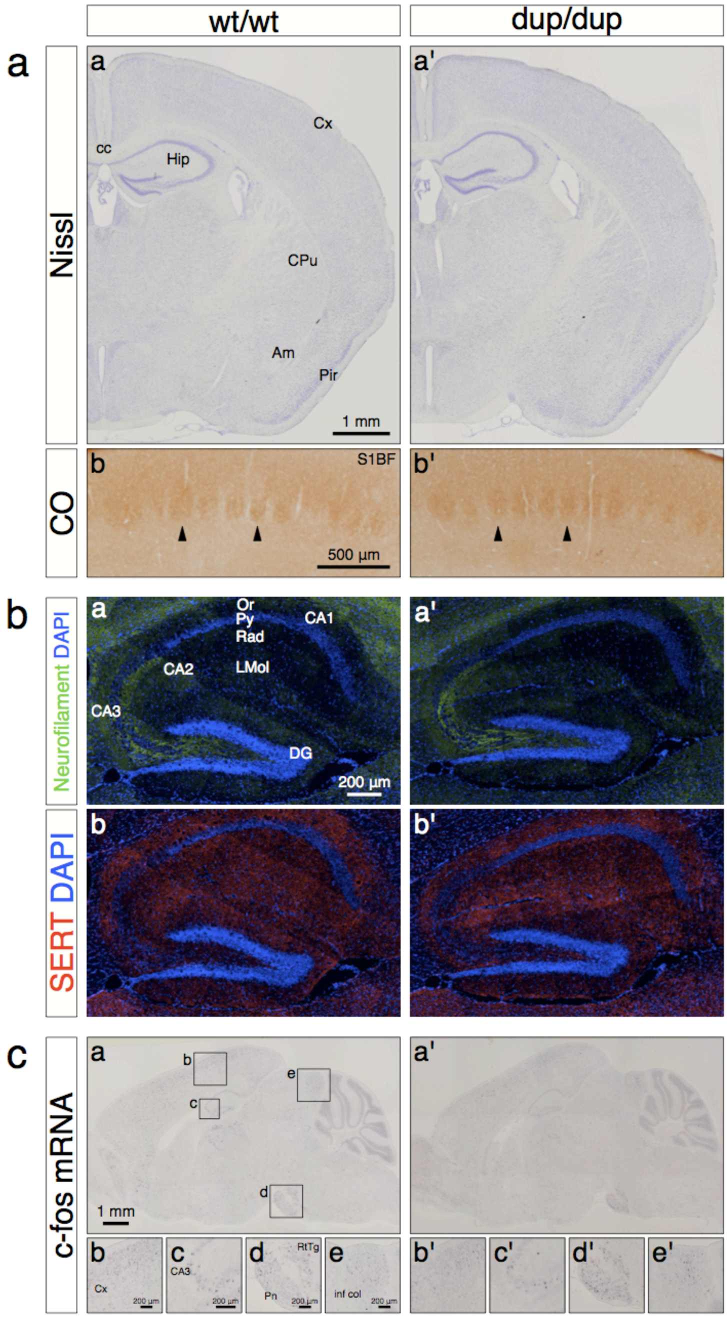

Figure S2. Macroscopically normal brain organization of the Pcdhα$^{dup(2-c2)/dup(2-c2)}$ mice. (a) Cortical sections from 4-week-old wild-type (wt/wt) and Pcdhα$^{dup(2-c2)/dup(2-c2)}$ (dup/dup) mice were stained with Nissl (a and a') and cytochrome oxidase (CO) (b and b'). Normal brain structures and barrels were visible in the Pcdhα$^{dup(2-c2)/dup(2-c2)}$ mice. The barrels in the primary somatosensory cortex (S1BF) layer IV are visualized as blobs of increased CO activity (arrowheads point to individual barrels). (b) Immunostaining for neurofilament and SERT in sagittal hippocampal sections from 4-week-old mice. Neural pathway and serotonergic neurons showed no obvious differences between the genotypes. (c) *In situ* hybridization analysis of the

c-fos mRNA expression in sagittal sections from 4-week-old mice. Expression of *c-fos*, well-known marker for neuronal activation, showed no obvious differences between the genotypes. Am, amygdala; cc, corpus callosum; CPu, caudate putamen; Cx, cerebral cortex; Hip, hippocampus; Pir, piriform cortex. CA1, hippocampal CA1; CA2, hippocampal CA2; CA3, hippocampal CA3; DG, dentate gyrus; LMol, lacunosum moleculare; Or, oriens layer hippocampus; Py, pyramidal cell hippocampus; Rad, radiatum layer hippocampus; inf col, inferior colliculus; Pn, pontine nuclei; RtTg, reticulotegmental nu pons.

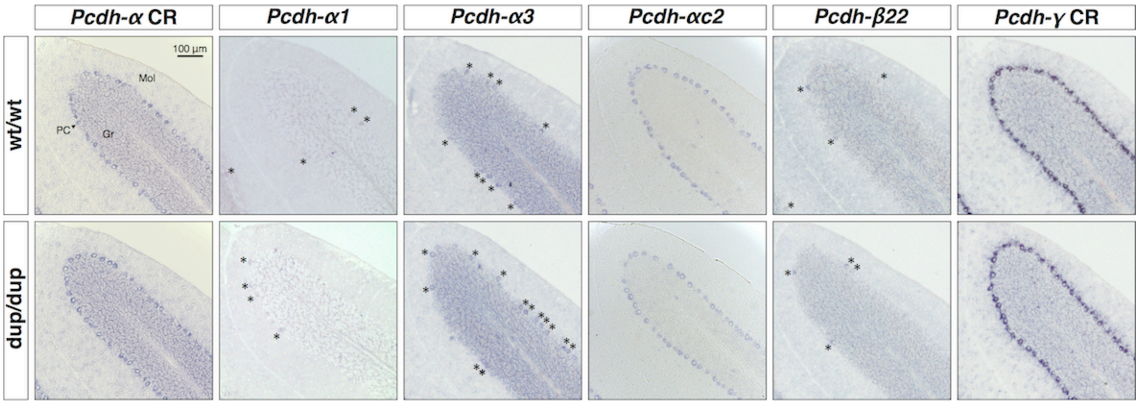

Figure S3. *In situ* hybridization analysis of *Pcdh* genes in the Pcdh$\alpha^{\mathrm{dup(2\text{-}c2)/dup(2\text{-}c2)}}$ cerebellum. Distribution of the *Pcdh-α* CR, *-α1*, *-α3, -αc2, -β22,* and *-γ* CR transcripts in the 6th cerebellar lobules of 4-week-old wild-type (wt/wt) and Pcdh$\alpha^{\mathrm{dup(2\text{-}c2)/dup(2\text{-}c2)}}$ (dup/dup) mice, by *in situ* hybridization. The photographs for the *Pcdh-α* CR are the same as in Figure 1G (right). Anterior is to the left, posterior to the right. All probes showed similar staining patterns between the wild-type and Pcdh$\alpha^{\mathrm{dup(2\text{-}c2)/dup(2\text{-}c2)}}$ mice. Asterisks indicate signal-positive Purkinje cells except for *Pcdh-α* CR*, -αc2,* and *-γ* CR. Gra, granule cell layer; Mol, molecular layer; Pur, Purkinje cell layer.

Supplementary Tables

Supplementary Table S1. Genotypes of progeny from Pcdh$\alpha^{wt/dup(2\text{-}c2)}$ intercrosses

Gender	No. (%) of offspring with each genotype			total
	wt/wt	wt/dup	dup/dup	
Male	52 (22)	117 (50)	64 (27)	233
Female	61 (24)	125 (50)	63 (25)	249

Supplementary Table S2. List of primers

Name	Sequence (5' > 3')	Purpose
**For generation of the SR allele**		
CP1proA-F	GAATTAGTGGTTAGAACCTCACCC	Probe A
CP1proA-R	TAGGACCAACCTAACCACAAGACCC	Probe A
C1proB-F	AAGGTCACTGCCTAATCTGG	Probe B
C1proB-R	CAAATCTGGCCATCCAAAGG	Probe B
C1H-F	cccaagcttggaatgcggccgctaAGCCACGCAAACATGATTGTGGG	SR targeting
C1H-R	acgcgtcgacgGTCGCAGAACTCTAGATGCAGG	SR targeting
C1F-F	acgcgtcgacgTGGTCCTTGAAGAACTTCCTCC	SR targeting
C1F-R	ctagctagcGCTTTCAAACAACAAGGCAGCC	SR targeting
SRC-F	GGAAGTCCATTTCATTTAGTCTGTAGG	SR targeting
SRC-R	ggaattccatcgatcgAATATGGCACCTGAAGACTGGTGGG	SR targeting
SRD-F	cgggatcccgAATGACAGTTTGTGGGCAGAGGG	SR targeting
SRD-R	cgggatcccgccatcgatggATAACTTCGTATAGCATACATTAT ACGAAGTTATTTCCAAGGAGCTCTGGGGGTACTGG	SR targeting (add loxP)
C1D-F	cgggatcccgAGAGCTCCTTGGAACTGTACCACC	SR targeting
C1D-R	aaaagcggccgcaacgctcgagcgGAAAGTACAGAAACCAGCCTGATCCC	SR targeting
**For genotyping the mutant mice**		
Cre-F	GCAGAACCTGAAGATGTTCGCGAT	Sycp1-Cre
Cre-R	AGGTATCTCTGACCAGAGTCATCC	Sycp1-Cre
G16F	GGCTATCCTGTGCTACAGAAC	G16Neo
G16R1	CCAATTAATATTTGAGATTCATCCC	G16Neo
G16R2	GTTGTACTCCAGCTTGTGCC	G16Neo
SR134F	ATTCCTGGTACTGGCATTCC	SR
SR454R	TACTTAGGGGTGCTGTGAGG	SR
SR202F	TGACCCCATCACTCCAGTGG	dup(2-c2)
IR/DR1.0F	AATTGGAGGTGTACCTGTGG	dup(2-c2)
CNR3376F	GACATATGATGGCCATCCTC	dup(2-c2)
CNR4077R	AAGTCTGCTCACTGTATGC	dup(2-c2) followed by *Taq*I digestion
**For real-time RT-PCR**		

CR2-3'F	CAGTGGCCAACAGTATCCAG	Pcdh-α CR
C3-5'	AGGTCCAGCTGTTGCTGTTG	Pcdh-α CR
Pcdha1-realF	CCCAGGTTTGAACATAGC	Pcdh-α1
Pcdha3-realF	CCATGCCCAGTTAATCAAG	Pcdh-α3
Pcdha5-realF	GGTGTTGGTTGGAACCTC	Pcdh-α5
Pcdha6-realF	CTAGTGCGGTAGGGAGC	Pcdh-α6
Pcdha7-realF	TAAGGTGTCAGCTGGAGCC	Pcdh-α7
Pcdha9-realF	TACACCTTGCCCAGTGG	Pcdh-α9
Pcdha10-realF	CCTGGTTTGGGTTCTGG	Pcdh-α10
Pcdha12-realF	AGTCTCCCTCCTGTGTTAGG	Pcdh-α12
Pcdhac2-realF	CAACAGGCAACTCACCG	Pcdh-αc2
Pcdha 2.3R	CGAGGCAGAGTAGCGCC	Pcdh-αs (common reverse)
Wdr55-realF	GATCCGTGATACCCCAGAAGAC	Wdr55
Wdr55-realR	CCTTGGTTTCTCCCTCCTGAC	Wdr55
Dnd1-realF	TCTGCGCCCTCTCAGATTG	Dnd1
Dnd1-realR	GGAAGTGCTGCTTTAGGTCTGG	Dnd1
Hars1-realF	GGAGCGGATCTTTTCCATTGTAG	Hars
Hars1-realR	CTTGATCCCAGCATCCCATAAC	Hars
Zmat2-realF	AAGGATGAAGGAGCTCAGAGAAGA	Zmat2
Zmat2-realR	AAGCCAGAGAAACCCATCACA	Zmat2
Vault-realF	CAGCTTTAGCTCAGCGGTTACTTC	Vault RNA
Vault-realR	GGTAAGTGGTTGGTTGTGTTGTTC	Vault RNA
Pcdhb2-realF	GGTGAGGATATACCGAGTCTAG	Pcdh-β2
Pcdhb2-realR	CCAGGAACATGCAAAGGATTAGAG	Pcdh-β2
Pcdhb3-realF	CTCTCTTGGTCCTGGTAAACTTC	Pcdh-β3
Pcdhb3-realR	GGGATACCATGGCTTGAATTACTG	Pcdh-β3
Pcdhb16-realF	GTTCTGGGATGGTTTGGAAATGTAC	Pcdh-β16
Pcdhb16-realR	GACCTCGTTGTGTTTGAGCATTG	Pcdh-β16
Pcdhb19-realF	TGCCAGCGCTTGTAATAAGATGG	Pcdh-β19
Pcdhb19-realR	TGTTGGAAAAGACAGGTGGGAAG	Pcdh-β19
Pcdhb22-realF	AACTATGGTAGGCAACCAGATGATC	Pcdh-β22
Pcdhb22-realR	GAATACAGAGAGCGAAATGTGACG	Pcdh-β22
Slc25a2-realF	ATGTCACCCAGGGTTCTGTC	Slc25a2
Slc25a2-realR	AGTCGTTCAGCTCTGCATTCTG	Slc25a2
Taf7-realF	AATCCGACGAGCAACACCAAG	Taf7
Taf7-realR	GGTTTCTTGGAGCTTGCCTTTC	Taf7
gamma cp1 realF	CTGGCGTTTCTCTCAAGCCC	Pcdh-γ CR
Pcdhg constant R2	CATGGCTTGCAGCATCTCTG	Pcdh-γ CR
Diap1-realF	CAGTCTTCTAGAAGCTCTGCAGTC	Diap1
Diap1-realR	CTCCGAGGCTAGCAGAGATG	Diap1
Hdac3-realF	GTCCCGAGGAGAACTACAGCA	Hdac3
Hdac3-realR	GAACTCTTGGGGACACAGCA	Hdac3
B2m-realF	CCCTGGTCTTTCTGGTGCTT	B2m

B2m-realR	ATGTTCGGCTTCCCATTCTC	B2m

For SNP analyses

Pcdh-α3

v3+846F	GTCACTTGAAATCCTGTC	RT-PCR & sequencing
Pcdha 2.3R	CGAGGCAGAGTAGCGCC	RT-PCR
CNR+1.4R	ACCAGCGAGTAGGACACC	colony PCR

Pcdh-α5

v5+1704F	TCAGGGTAGCGGAGGAAC	RT-PCR & sequencing
Pcdha 2.3R	CGAGGCAGAGTAGCGCC	RT-PCR
	Colony PCR using T7 & M13-20, then *Tsp45*I digestion	

Pcdh-α6

v6-ATG-F	AATGGATTTTACCACTGAAG	RT-PCR & sequencing
Pcdha 2.3R	CGAGGCAGAGTAGCGCC	RT-PCR
CNR+0.3R	TGTCCACGATCACCTCCA	colony PCR followed by *Bsa*HI digestion

Pcdh-α7

v7+1061F	CCTTGTCTCTCCCAGTATCC	RT-PCR & sequencing
Pcdha 2.3R	CGAGGCAGAGTAGCGCC	RT-PCR
CNR+1.4R	ACCAGCGAGTAGGACACC	

Pcdh-α9

v9+649F	GGCAAAGGCAAATTTCCAGTG	RT-PCR & sequencing
Pcdha 2.3R	CGAGGCAGAGTAGCGCC	RT-PCR
v09-4	ACGGTGCAGTGAGCAACCAT	colony PCR

Pcdh-α10

v10+403F	CCAACGACAGAAAAGAATCTC	RT-PCR & sequencing
Pcdha 2.3R	CGAGGCAGAGTAGCGCC	RT-PCR
CNR+0.8R	GACCATTTGAACCTTCGTC	colony PCR followed by *Bst*YI digestion

Pcdh-α12

v12-ATG-F	ATGCTATTATTCTCGCAAAGC	RT-PCR & sequencing
Pcdha 2.3R	CGAGGCAGAGTAGCGCC	RT-PCR
CNR+0.3R	TGTCCACGATCACCTCCA	colony PCR & sequencing

Pcdh-αc2

C2+466F	GTAAGCGAATCCGTGGCACCTGGAG	RT-PCR
CR12-R	CCTCCTCCAGGTGCACAGAGCTG	RT-PCR
C2+978R	CTCCTCATAATCCAGAGTCC	colony PCR

C2+542F	TGCAGACCTACGAGCTCAG	sequencing

Common primers for colony PCR

T7	GTAATACGACTCACTATAGGGC
M13-20	GTAAAACGACGGCCAGTG

For single-cell RT-PCR (*Pcdh-α3, -α5, -α7*, and *Pcp-2*)

Reverse transcription

CNR2596RS	GACTGTTTGGGGTTGCC
Pcp2 429R	GTTTTCAGGGGCCAGTGG

1st multiplex PCR

v1+1176F	TCATGTCCCTTTCAAGTTGGTGTCCAC
v3+1166F	AGGTTCCCTTCAAGCTGGTGTCCAC
v4+1252F	ATGTCCCCTTCAAGTTGGTGTCCAC
v5+1176F	CATCCCCTTCAAGCTGGTGTCCAC
v7+1175F	ATATCCCCTTCAAGCTGGTGTCCAC
v11+1170F	CAATGTCCCCTTCAAGATTGTATCCACC
v12+1176F	CCTCCCCTTCAAGCTGGTGTCCAC
CR12-R	CCTCCTCCAGGTGCACAGAGCTG
Pcp2 15F	TAGGGGCACTTCTGAGCC
Pcp2 429R	GTTTTCAGGGGCCAGTGG

2nd nested PCR

Pcdh-α3

a3sscF	GTGGATTCTGCCGGTAGG	PCR & sequencing
CNR2429RS	GTGCATGCCTGCTCTTA	
Pcdha 2.3R	CGAGGCAGAGTAGCGCC	sequencing
v3+1221F	CAGTGTCCTGGACCGAGAGAC	PCR & sequencing
v3+2061R	AAGCGAAGCCTCCCTACCG	
CNR+1.4R	ACCAGCGAGTAGGACACC	sequencing

Pcdh-α5

a5sscF	CAGGGTAGCGGAGGAACT	PCR & sequencing
CNR2429RS	GTGCATGCCTGCTCTTA	
Pcdha 2.3R	CGAGGCAGAGTAGCGCC	sequencing

Pcdh-α7

a7sscF	GTATCAAGAGCTTTGGTGCC	PCR & sequencing
CNR2429RS	GTGCATGCCTGCTCTTA	
Pcdha 2.3R	CGAGGCAGAGTAGCGCC	sequencing
v7+1231F	AGTGCTCTGGACCGAGAGAC	PCR & sequencing

v7+2080R	CCAGCCTCTGATCCACTCTG	
CNR+1.4R	ACCAGCGAGTAGGACACC	sequencing

Pcp-2

Pcp2+71F	CTGGCAGGTTCACCGGAC	
Pcp2+411R	GAGGCCCAGGATGGCTAG	

For single-cell RT-PCR (*Pcdh-αc2* and *Pcp-2*)

Reverse transcription

CNR2596RS	GACTGTTTGGGGTTGCC
Pcp2 429R	GTTTTCAGGGGCCAGTGG

1st duplex PCR

C2+466F	GTAAGCGAATCCGTGGCACCTGGAG
CR12-R	CCTCCTCCAGGTGCACAGAGCTG
Pcp2 15F	TAGGGGCACTTCTGAGCC
Pcp2 429R	GTTTTCAGGGGCCAGTGG

2nd nested PCR

Pcdh-αc2

C2+498F	TCACATAGAGAGCGCACAGG	PCR
CNR2429RS	GTGCATGCCTGCTCTTA	PCR
C2+542F	TGCAGACCTACGAGCTCAG	sequencing
C2+978R	CTCCTCATAATCCAGAGTCC	sequencing

Pcp-2

Pcp2+71F	CTGGCAGGTTCACCGGAC
Pcp2+411R	GAGGCCCAGGATGGCTAG

For methylation analysis using bisulfite sequencing

v1Me-429F	GATATATGTATGTAATTGTTAGAGTGGGGTG	Pcdh-α1
v1Me+6R	CAACATTACAAAAATACTACACTTTCC	Pcdh-α1
v1Me-28R	CTATCACACCAAAATCATACTTTACACCTTC	Pcdh-α1
v6Me-34F	TTTTTTAATGGAAATAAAGTAAGAAGTATTTGAAA	Pcdh-α6
v6Me+246R	ACCATTCTACAAATTTACCTCCAAAAAATC	Pcdh-α6
v6Me-16F	GTAAGAAGTATTTGAAATGGATTTTATTATTGAAG	Pcdh-α6
v12Me-4291F	AGGGTATAAAATGAGAATGTAGATTGGATT	Pcdh-α12(up)
v12Me-3920R	CCTATCTCTTAATAACTTTTATCCTTTCATAAACTT	Pcdh-α12(up)
v12Me-3941R	TCCTTTCATAAACTTATATTTTAAAAAAAA	Pcdh-α12(up)
v12MeR-93F	GTATTAGAGAATTTATAGAGAGTTTTTTGAAGGAA	Pcdh-α12
v12MeR-476R	CATAACTATAAAAACTAAAAACCTAACACAAC	Pcdh-α12

v12MeR-117F	TTTTGAAGGAAGTGATTTTTTTTGAAAAGA	Pcdh-α12
v12Me+2000F	TGTTTTTGGTGGAGAATGGTTAAGTATTTAA	Pcdh-α12(3')
v12Me+2391R	TTTCAAATATTCTACCTACCTTTCTCTCTAACATAT	Pcdh-α12(3')
v12Me+2013F	GAATGGTTAAGTATTTAAGATATTTTTTTT	Pcdh-α12(3')
v12Me+2382R	TTCTACCTACCTTTCTCTCTAACATATATCCTCTAC	Pcdh-α12(3')
C1-Me1stF	CCCCAAAAACCTACCTTATCACCCCATAT	Pcdh-αc1
C1-Me2ndF	AACCAACAATAACCAACCAACAACCACA	Pcdh-αc1
C1-MeR	TGTTTTGAA TTTTTGGAGAGGAGATTGG	Pcdh-αc1
C2Me+87F	ATTGTTGTTGGTGTTGTTGTTAGGTTTAG	Pcdh-αc2
C2Me+429R	ATTATCATTAATATCCAATATTTCCACCT	Pcdh-αc2
C2Me+391R	TCACTACCACAAAATTATAAACCAACAC	Pcdh-αc2

For methylation analysis of *Pcdh-αc2* by HpaII digestion

C2+117F	GGCCTCCCAGCTACGATACTC	PCR
C2+693R	CCCATCCACCGCAGTGAGAAC	PCR
C2+542F	TGCAGACCTACGAGCTCAG	sequencing

Supplementary Table S3. List of SNPs analyzed in single-cell RT-PCR analysis

Target gene	SNP position from B6 start codon	5'(B6)/3'(CBA)/JF1
Pcdh-α3	1260	T/T/G
	1278	C/C/T
	1291	T/C/T
	1338	T/T/C
	1383	A/A/G
	1404	C/C/A
	1424	C/C/T
	1425	A/A/G
	1437	G/G/A
	1443	A/A/C
	2175	T/T/G
	2226	T/T/C
	2365	A/A/G
Pcdh-α5	1832	C/T/C
	1867	C/C/T
Pcdh-α6	201	G/T/G
Pcdh-α7	1338	T/C/C
	1515	G/G/T
	1554	C/C/T
	1557	T/T/G
	1587	A/A/G
	1942	T/T/C

	2064	A/A/C
Pcdh-α9	862	A/C/C
Pcdh-α10	451	C/T/T
Pcdh-α12	-4213	A/del/?
	-380	CAx5/del/?
	52	T/C/C
Pcdh-αc2	667	T/C/C

Supplemental References

1. Liu, P., Jenkins, N.A. & Copeland, N.G. A highly efficient recombineering-based method for generating conditional knockout mutations. *Genome Res* **13**, 476-484 (2003).
2. Yokota, S. *et al*. Identification of the cluster control region for the Protocadherin-{beta} genes located beyond the Protocadherin-{gamma} cluster. *J Biol Chem* (2011).
3. Mishina, M. & Sakimura, K. Conditional gene targeting on the pure C57BL/6 genetic background. *Neurosci Res* **58**, 105-112 (2007).
4. Takeuchi, T. *et al*. Flp recombinase transgenic mice of C57BL/6 strain for conditional gene targeting. *Biochem Biophys Res Commun* **293**, 953-957 (2002).
5. Esumi, S., Kaneko, R., Kawamura, Y. & Yagi, T. Split single-cell RT-PCR analysis of Purkinje cells. *Nat Protoc* **1**, 2143-2151 (2006).
6. Hirano, K. *et al*. Single-neuron diversity generated by Protocadherin-beta cluster in mouse central and peripheral nervous systems. *Front Mol Neurosci* **5**, 90 (2012).
7. Kaneko, R. *et al*. Allelic gene regulation of Pcdh-alpha and Pcdh-gamma clusters involving both monoallelic and biallelic expression in single Purkinje cells. *J Biol Chem* **281**, 30551-30560 (2006).
8. Watakabe, A. *et al*. Comparative analysis of layer-specific genes in Mammalian neocortex. *Cereb Cortex* **17**, 1918-1933 (2007).
9. Watakabe, A., Komatsu, Y., Ohsawa, S. & Yamamori, T. Fluorescent in situ hybridization technique for cell type identification and characterization in the central nervous system. *Methods* **52**, 367-374 (2010).
10. Katori, S. *et al*. Protocadherin-alpha family is required for serotonergic projections to appropriately innervate target brain areas. *J Neurosci* **29**, 9137-9147 (2009).
11. Noguchi, Y. *et al*. Total expression and dual gene-regulatory mechanisms maintained in deletions and duplications of the Pcdha cluster. *J Biol Chem* **284**, 32002-32014 (2009).

12. Takahata, T., Hashikawa, T., Higo, N., Tochitani, S. & Yamamori, T. Difference in sensory dependence of occ1/Follistatin-related protein expression between macaques and mice. *Journal of chemical neuroanatomy* **35**, 146-157 (2008).
